# Caffeinated Energy Drink Induced Ventricular Fibrillation: The Price for Overexcitement

**DOI:** 10.7759/cureus.6358

**Published:** 2019-12-11

**Authors:** Hesham Osman, Sadeq Tabatabai, Mahmoud Korashy, Mohamed Hussein

**Affiliations:** 1 Cardiology, Dubai Hospital, Dubai, ARE

**Keywords:** energy drink, in-hospital cardiac arrest, ventricular fibrillation

## Abstract

An otherwise healthy 32-year-old man had an in-hospital cardiac arrest with ventricular fibrillation after a few days of consuming 48 cans of alcohol-mixed energy drinks (EDs) (250-mL per can ). He had collapsed shortly after presenting to the emergency room with complaints of lack of sleep and palpitations. Normal cardiac rhythm was restored by biphasic direct current (D/C) shock. EDs generally contain mainly caffeine, taurine, and other ingredients. Especially in high doses, caffeine can cause palpitations and ventricular arrhythmias.

## Introduction

Energy drinks (EDs) are usually consumed by adolescents and young adults. They are marketed for the claim of their ability to supply energy, heighten mental alertness, and enhance physical performance. They usually contain caffeine, sugar, taurine, vitamins, and other ingredients like alcohol in some of them. The safety of EDs remains questionable due to a number of case reports in the past decade that have associated EDs intake with adverse cardiac events including death, cardiac arrest, atrial and ventricular arrhythmia and myocardial infarction [1-6].

## Case presentation

**Figure 1:**
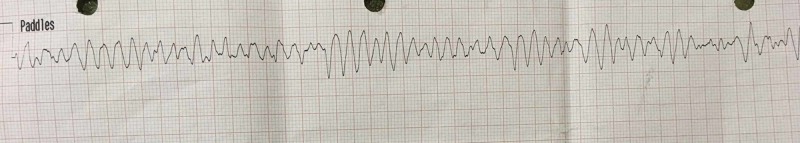
Cardiac monitor tracing showing ventricular fibrillation

**Figure 2:**
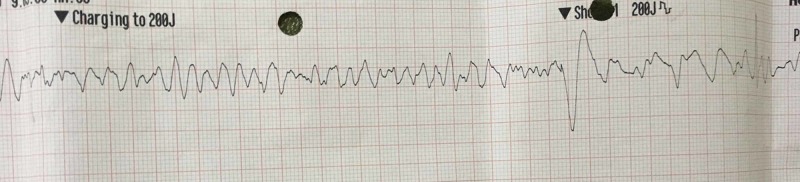
Cardiac monitor tracing during defibrillator shock

An otherwise healthy 32-year-old male was admitted to the emergency department at Dubai Hospital in August 2019 and sustained an in-hospital cardiac arrest soon after initial assessment. The patient had consumed 48 cans of 250-mL ED (XXL, containing a mixture of caffeine, vodka, and taurine) over the past three days. He collapsed shortly after presenting to the emergency room (ER) with complaints of lack of sleep and palpitations which started the night before the day of presentation to the ER. The cardiac monitor showed ventricular fibrillation and by biphasic direct current (D/C) shock, normal cardiac rhythm was restored (Figures 1-2).

As per his medical history, he was fit and well without any chronic illnesses and was not on any medications. On arrival at the ER, he was conscious and oriented, not in distress but looked anxious. Suddenly, he had visible convulsions and became unresponsive with up rolling of the eyes. Cardiac monitor showed ventricular fibrillation for which he received one D/C 200 J shock and normal sinus rhythm was restored after very short cardiopulmonary resuscitation (CPR). He recovered soon after defibrillation and was found to be fully alert and cooperative with spontaneous breathing (intubation and mechanical ventilation were not required). 

Initially, the patient was admitted to the coronary care unit (CCU) and was discharged after three days alive and well. Rest of his hospital stay was uneventful and he remained asymptomatic. He was contacted in November 2019 and found to be alive and healthy with no further cardiac events.

**Figure 3:**
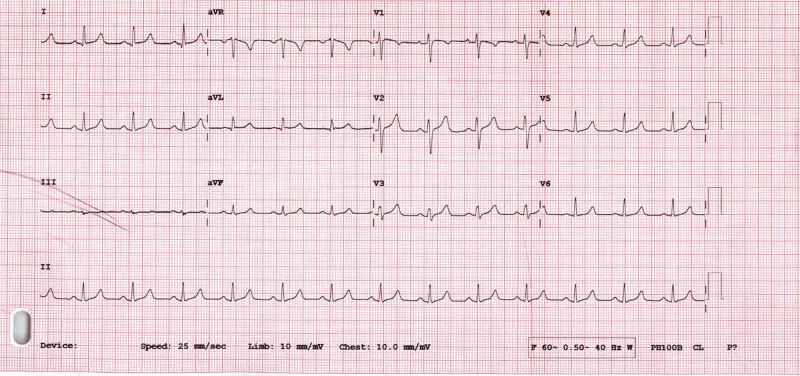
Resting electrocardiogram (ECG) after defibrillator shock

**Figure 4:**
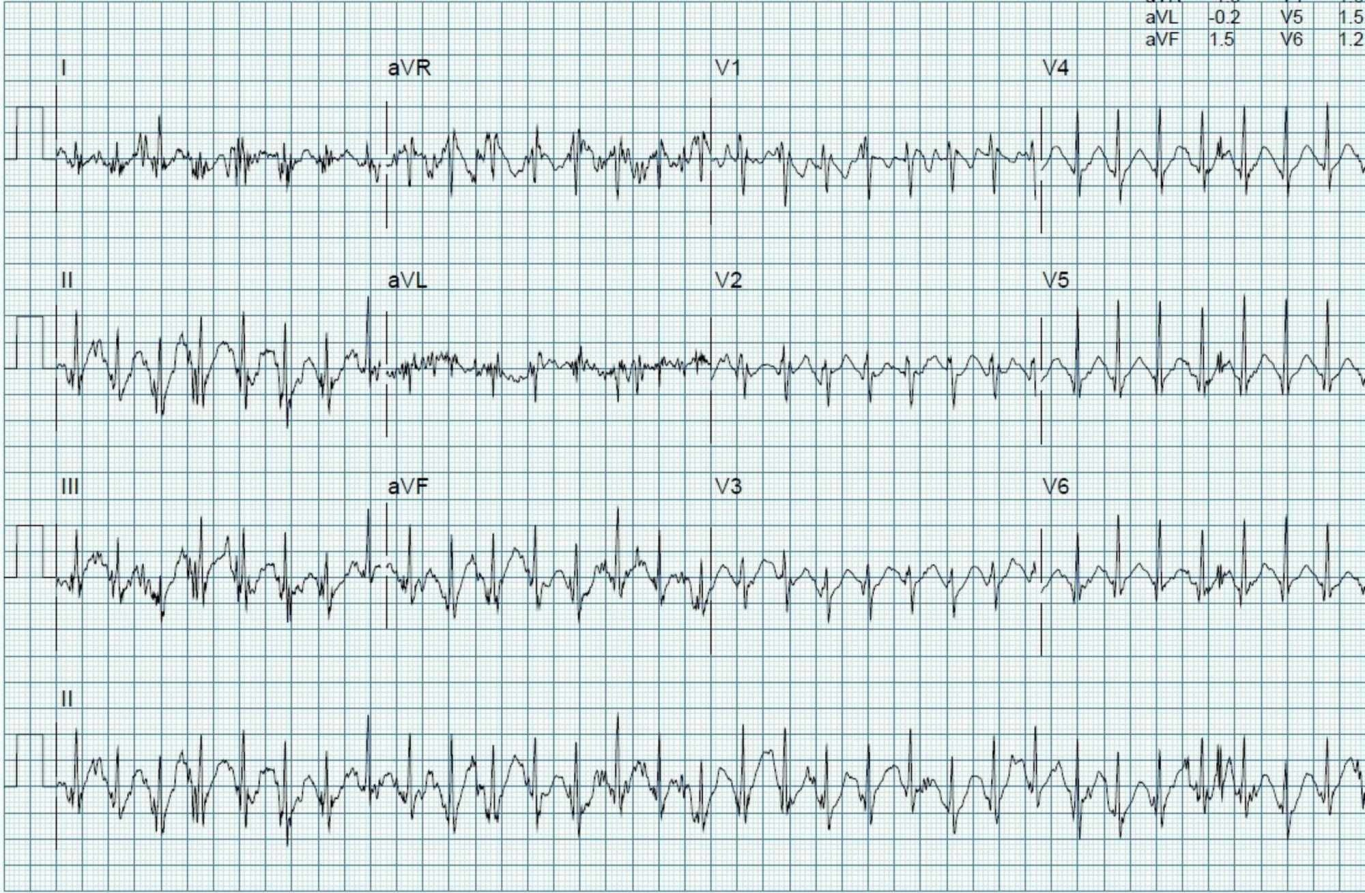
Peak treadmill exercise stress electrocardiogram (ECG) test

His initial vital signs showed blood pressure of 100/83 mmHg, heart rate 90 bpm and oxygen saturation of 100%. His clinical examination was unremarkable. Initial laboratory results showed normal serum electrolytes, creatinine, and complete blood counts. His peak creatine phosphokinase (CPK), creatine kinase-MB (CKMB) and high sensitive troponin T level were 118 U/L, 17 U/L and 30 ng/L respectively. An electrocardiogram (ECG) in sinus rhythm showed no electrocardiographic changes and no ST-segment changes (Figure 3). Echocardiogram showed normal study with normal left ventricular systolic and diastolic function, without any regional wall motion abnormalities, and there was no evidence of pulmonary hypertension. A treadmill exercise stress ECG test showed no evidence of exercise-induced myocardial ischemia or arrhythmias. He managed to do a total exercise time of 10:58 min on the Bruce protocol with maximum heart rate achieved of 188 bpm and workload achieved of 12.8 metabolic equivalents (METs) at the peak of exercise (Figure 4).

## Discussion

EDs are commonly used as a dietary supplement by young adults. They are often used as a source of energy in order to enhance physical and mental performance. Safety has been the biggest concern associated with consuming EDs. Over the past decade, it has been noticed that unexplained cardiovascular events (including death, cardiac arrest, atrial and ventricular arrhythmia, and myocardial infarction) in some young individuals developed after consuming EDs [1-5]. It is known that several stimulants are included in formulas of different EDs and the key ingredient in EDs is a high dose of caffeine, which is a methylxanthine, part of a chemical family that includes theophylline and aminophylline. More credible is the argument that EDs affect the cardiovascular conduction system and could easily lead to severe cardiovascular catastrophic events and arrest due to lethal arrhythmias and myocardial ischemia or infarction [6]. The arrhythmic complications of EDs are primarily attributed to caffeine [3]. There are increasing reports of adverse cardiovascular effects related to acute consumption of high dose of caffeine, particularly in the form of EDs including pro-arrhythmic effects, which are likely mediated by phosphodiesterase inhibition and increases in intracellular calcium release (concentration) and myofilament sensitivity [7]. Consumption of EDs in healthy people alters repolarization of the cardiac cycle possibly predisposing them to physiological responses that can lead to arrhythmias and other abnormal cardiac responses [8]. Even inherited cardiac channelopathies such as long QT and Brugada syndrome can be unmasked and provoked in a patient by the EDs that he consumed [6,9]. Myocardial ischemia due to either coronary spasm or thrombosis may also be caused by excessive EDs consumption. This is likely mediated by increased platelet aggregation, blood pressure elevation, and endothelial dysfunction [10].

Our patient presented to the ER with a history of lack of sleep after the overuse of EDs. He had in-hospital cardiac arrest with ventricular fibrillation and by biphasic D/C shock, normal cardiac rhythm was restored. The ED consumed by our patient contains 25 mg/100 ml of caffeine (equivalent to 62.5 mg per can) in combination with other ingredients such as taurine (10.2%) mixed with vodka. He drank 16 cans per day on average over three consecutive days, up to 1000 mg daily dose of caffeine, which is considered as a high to very high daily dose of caffeine [11]. There are case reports of ventricular arrhythmias and sudden cardiac death secondary to EDs. These were reported in both patients with and without structural heart disease [5]. Ehlers et al. demonstrated that heavy caffeine consumption may increase the risk of adverse effects including, nervousness, anxiety, insomnia, heart palpitations, and tachycardia [12]. Administration of high dose of caffeine in a murine model resulted in sympathetic over activity with ventricular ectopy culminating in ventricular fibrillation [7,13]. We have given due consideration to the possibility of acute coronary syndrome, but we felt that this diagnosis is unlikely based on a careful history, physical examination, laboratory tests, normal resting ECG, echocardiography, and negative stress ECG.

## Conclusions

In summary, our patient survived an in-hospital cardiac arrest following excessive consumption of alcohol-mixed EDs. The cardiac arrest is linked to the overuse of caffeinated alcohol-mixed ED that he consumed. Public awareness regarding the possible lethal cardiac consequences of the overconsumption of caffeinated EDs is probably indicated.
